# Challenges and opportunities in transitional care process in Behçet’s syndrome

**DOI:** 10.3389/fmed.2024.1456063

**Published:** 2024-09-18

**Authors:** Federica Di Cianni, Maria Vincenza Mastrolia, Edoardo Biancalana, Diana Marinello, Giacomo Emmi, Marta Mosca, Gabriele Simonini, Rosaria Talarico

**Affiliations:** ^1^Rheumatology Unit, Azienda Ospedaliero-Universitaria Pisana, Pisa, Italy; ^2^Department of Medical Biotechnologies, University of Siena, Siena, Italy; ^3^Rheumatology Unit, ERN ReCONNET Center, Meyer Children’s Hospital IRCCS, Florence, Italy; ^4^NEUROFARBA Department, University of Florence, Florence, Italy; ^5^Department of Experimental and Clinical Medicine, University of Florence, Florence, Italy; ^6^Department of Medical, Surgery and Health Sciences, University of Trieste, Italy, and Clinical Medicine and Rheumatology Unit, Cattinara University Hospital, Trieste, Italy; ^7^Centre for Inflammatory Diseases, Monash University Department of Medicine Monash Medical Centre, Melbourne, VIC, Australia

**Keywords:** Behçet’s syndrome, pediatric rheumatology, transitional care, review, patient centred care

## Abstract

Behçet’s syndrome (BS) is a rare chronic multi-systemic inflammatory disorder that usually involves adults between third and fourth decades of life, while pediatric and juvenile onset are relatively rare. BS young patients (YP) often develop a full-blown disease late after onset, requiring careful diagnostic workup and regular follow-up while they grow up. In this regard, the purpose of transitional programs is to ensure continuous high-quality care to YP with chronic conditions, providing them with the skills necessary to become independent and empowered adults able to chronically self-manage their disease. EULAR/PReS released the first set of standards and recommendations for transitional care (TC) of YP with juvenile-onset rheumatic diseases, but the appropriate timing for transition, the tools to evaluate patients’ readiness, and indicators of transition plans effectiveness still need to be identified. Although little is known regarding TC in BS, it is easy to assume that BS YP will benefit from developmentally and disease-specifically appropriate transition plans, which may promote continuity of care, improve perceived quality of life and prevent poor disease outcomes. This perspective article discusses the key concepts and the goals of TC, addressing the potential challenges and opportunities of TC for YP with BS in clinical practice.

## Introduction

Adolescence and young adulthood represent a crucial and unique phase in young patients’ (YP) life, who need education, support, and guide to grow as aware and responsible adults on health matter. This principle applies even more to YP with chronic conditions and, more specifically, with child-onset chronic rheumatic diseases (RD) since they need to acquire further skills and knowledge to independently self-manage their illness lifelong (1, 2). The need for a transition programme from pediatric to adult medical care for YP with chronic diseases originates from the evidence that they have poorer outcomes including increased rates of emergency room visits and hospitalizations, lack of adherence to treatments, loss to follow-up (3–5), poor social/working functioning (5, 6), and higher health-care costs (3–6). These complications are also well described for patients affected by RD, especially systemic lupus erythematosus (SLE) and juvenile idiopathic arthritis (JIA), showing that these patients often enter adulthood with active disease or develop flares as adults, require complex immunosuppressive therapy regimens, and are at risk of early organ damage and morbidity (2, 4, 7–9). Less is known about the outcome of pediatric-onset Behçet’s syndrome (BS), a rare chronic multi-systemic inflammatory disorder presenting with a highly heterogeneous clinical spectrum, which usually involves adults between third and fourth decades of life, although the onset potentially can occur at any age (10). Many cohort studies reported children with BS having overall lower severity score and activity index compared with adult patients (11–13). However, clinical expression of pediatric BS shows pronounced geographical and gender distribution, as well as adult-onset BS, and pediatric patients usually take longer to develop a full-blown disease phenotype, therefore they should be followed-up carefully over time to acknowledge disease spectrum and evolution (11). Moreover, BS has a dramatic impact on patients’ quality of life (QOL) (14), given the chronic course and the potentially severe organ involvement of the disease, thus it seems clear that YP with BS would greatly benefit from developmentally and disease-specifically appropriate transition plans. These would ensure a continuous high-quality care during and after adolescence, aimed at minimizing the development of disease complications. Nonetheless, child-onset BS is an understudied topic and transitional care is even more. In the present perspective article adult-onset and pediatric-onset BS will be explored, and opportunities and challenges of TC in clinical practice will be accordingly discussed.

## What is peculiar of adult Behçet’s syndrome?

BS is a systemic vasculitis of unknown origin, which may affect any size of the blood vessels and involves potentially any organ systems. BS pathogenesis is still unclear but the most accepted hypothesis is that various environmental and/or infectious triggers may start the activation of inflammatory pathways in subjects with genetic susceptibility (15, 16). The disease usually develops in young-adults, but pediatric-and juvenile-onset is reported in up to 15–20% of cases (11, 17). The course of disease is relapsing-retting and its burden tends to be heavier in the early years after onset, while the degree of disease activity decreases over time (15). Oral and genital recurrent ulcerations are the clinical hallmarks of BS and commonly develop at disease onset or early during the disease course (15, 18, 19). Papulopustular skin lesions and erythema nodosum are the second most frequent clinical expression of BS, and are equally seen in both genders. Articular involvement affects up to 80% of patients of both genders, and usually presents as a mono-or oligo-arthritis of knees, ankles and wrists (15, 18, 19).

Ocular and vascular involvement usually develop early in the disease course, in contrast to gastro-intestinal (GI) and neurologic manifestations, but each of them is potentially organ-threatening and associated to high rates of morbidity and mortality (15, 20). The most frequent ocular manifestation are panuveitis or posterior uveitis, often bilaterally. On the contrary, anterior uveitis is less common and mainly seen in female patients. Retinal vasculitis can be seen in association with posterior uveitis in half of the cases and may indicate a severe involvement (19). Main vascular manifestation is represented by deep vein thrombosis (DVT) of upper and lower limbs, although atypical sites such as vena cava, hepatic veins with Budd-Chiari syndrome and cerebral venous sinuses are quite specific of BS. Arterial aneurysms are also specific of BS, especially at sites like pulmonary, peripheral and visceral arteries (15, 19). Central nervous system (CNS) involvement shows a male predominance and presents with an acute and sub-acute onset of headache, focal symptoms like hemiparesis, pyramidal signs, and neuro-cognitive/psychiatric disorders. The parenchymal inflammatory lesions usually affect brainstem, telencephalic-diencephalic junction and basal ganglia and, more occasionally, cerebellum and spinal cord. Together with vascular involvement, parenchymal neurological manifestations are the leading cause of BS-related mortality (15, 19). Finally, GI involvement is infrequent on a global scale, but it shows high prevalence in Far-East Asia (15). Abdominal pain, diarrhea and vomiting are the most common clinical features regarding GI involvement, but haemorrhage and perforation rarely may occur. BS causes round ulcers most commonly in the mucosa of terminal ileum, similarly to Crohn’s disease, making endoscopy necessary—although not always sufficient-to distinguish the two conditions (15, 19).

The prevalence of the various manifestations, the clinical course and the long-term outcomes follow a peculiar geographical distribution in BS and vary according to patients’ ethnical background, gender, and age of onset. Such heterogeneity may be explained by the existence of numerous etiopathogenetic mechanisms underlying the different clinical presentations, which still need to be clarified (19, 21). Furthermore, disease manifestations may overlap during the course of disease, resulting in a variable spectrum of disease which distinguishes different patients, but may also represent different phases of the disease in the same individual (16, 22). Indeed, recently the concept has emerged that BS may not be a single clinical entity, but a complex multi-system disorder resulting in different clinical phenotypes (16, 19, 21, 22).

Given the absence of pathognomonic laboratory or radiological tests, BS diagnosis is only supported by clinical criteria that require the exclusion of other diagnoses. Several international groups have attempted to propose set of clinical criteria for classification/diagnosis of BS in adult patients. The most currently used set are those published in 1990 by the International Study Group (ISG) (23) and those published in 2014 (New international criteria for BS—ICBS) (12). ISG criteria considered oral aphthosis as mandatory for BS classification, in opposite to ICBS, which added neurological and vascular manifestations amongst the items. For ISG and ICBD criteria, sensitivity and specificity of are quite high, but the satisfaction of either these criteria is not sufficient to formulate a definitive diagnosis of BS, and a full clinical evaluation to exclude differential diagnoses is still necessary.

According to current guidelines (24, 25), a multidisciplinary approach is necessary in the management of BS, and treatment should aim at suppressing inflammatory exacerbations and preventing irreversible organ damage. Treatment choice should be personalized based on the patient characteristics and preferences. Albeit topical treatment may be useful for aphthosis and skin lesions, systemic treatment with colchicine is recommended to prevent muco-cutaneous lesions recurrency. Colchicine is also the initial treatment for BS arthritis, but immunosuppressants like azathioprine (AZA) or anti-tumor necrosis factor alpha (anti-TNFa) should be considered in chronic or refractory cases. In acute ocular attacks, topical therapy with glucocorticoids (GCs) and mydriatics should be promptly given, but systemic GCs may be associated with AZA or cyclosporine (CyA) in unresponsive patients and in case of posterior uveitis. Systemic GCs and AZA/CyA are recommended also for the management of the other major organ involvement (vascular, intestinal, and neurological). The use of anti-TNFa is usually limited to non-responsive patients, yet they are recommended as first choice in severe CNS or GI disease. Moreover, cyclophosphamide is recommended as first-line therapy when arterial aneurysms occur. Last, cyclosporine should be avoided in CNS involvement (24–26).

## What is peculiar of pediatric Behçet’s syndrome?

BS onset before the age of 16 is reported in 4 to 26% of cases (27). Pediatric BS is relatively rare in children, with a prevalence ranging from 0.2 to 10/100,000 children with a wide geographic variability, being more prevalent in the regions along the ancient Silk Road, including Middle East, Mediterranean countries and East Asia, as observed in adult patients (28). In most series, boys and girls are affected with equal frequency and the mean age of onset significantly varies between different studies, from 4.87 to 12.3 years (27–29). Diagnostic delay is approximately 3 years as patients often show an incomplete phenotype, and the full-blown disease may develop several years later (29).

Since the performance of ICBD and ISG criteria is not optimal in the pediatric setting, a set of criteria specifically designed for the pediatric BS patients has been recently proposed. In the classification criteria of pediatric Behçet disease (PED-BD), recurrent oral ulceration is included, although it is not mandatory, along with recurrent genital ulceration, skin, eye, neurological and vascular involvement. The presence of three out of six criteria classifies patients as having BS (30).

A familial clustering of BS is reported to have a higher prevalence in childhood and is known to be characterized by genetic anticipation, accounting for earlier disease onset in successive generations. According to literature estimates, the prevalence of familiar BS significantly varies among ethnic groups, being lower among Europeans (1%) as compared with other ethnicities (up to 18% in Turkish descents) (28, 31). BS familial clustering supports the idea of a genetic background sustaining the disease. While human leukocyte antigen (HLA)-B*51 is the allele most strongly associated with BS, genome-wide association studies have revealed genetic links with interleukin (IL)-10, IL-23R, IL-12RB2, C-C chemokine receptor (CCR) 1, signal transducer and activator of transcription (STAT) 4, MEFV, and toll-like receptor (32, 33).

The main manifestations of pediatric BS seem to be similar to those of adult patients with a high heterogeneity and variability according to geographic distribution. Furthermore, differences in the frequencies of some features have been found in pediatric patients (13, 34–36). An Israelian study by Krause et al. reported that arthralgia, neurologic and gastrointestinal involvement more frequently occurred in juvenile-onset BS (34). Differently, Zou et al. observed that, compared with adult BS patients, pediatric-diagnosed patients had a higher frequency of folliculitis, a lower frequency of arthralgia and panuveitis, and no cardiac lesions (35). Makmur et al. reported that a pediatric BS cohort was more likely to have gastrointestinal involvement and arthritis, and less likely to have eye involvement, skin and vascular involvement compared with adults (36). In a recent comparative study, we reported that articular manifestations was more common in the pediatric BS cohort, whereas venous vascular events were more frequent in the adult BS cohort (13).

In pediatric BS patients, oral ulcers often stand out as the most common and initially singular manifestation before other signs of the disease emerge over time (37). Conversely, genital ulcers are less prevalent, typically appearing after the onset of oral ulcers, and have been reported to be more frequent in females (38, 39). Additional skin lesions are observed in over 90% of BS children, encompassing conditions such as erythema nodosum, purpura, papulopustular lesions, ulcers, or folliculitis (40). Eye lesions are reported in 30 to 61% of BS children. The typical eye involvement is chronic relapsing bilateral posterior and anterior uveitis (40). Age seems to delineate the type of ocular involvement, with anterior uveitis being more prevalent in patients under 10 years old, whereas panuveitis is predominant in those over 10 years old. Moreover, ocular disease tends to be notably more frequent in boys (41).

Arthritis occurs in 50–75% of BS children, mostly affecting the large joints (knees, ankles, wrists, and elbows), usually presenting as oligoarticular and non-erosive, with potential development of sacroiliitis (37). The rate of vascular involvement in pediatric BS is reported to be 1.8 to 21%, even if the real prevalence during childhood is largely unknown (42, 43). Thrombosis represents the most frequent manifestation of vascular involvement and may occur at BS onset. Cerebral sinus veins represent the most frequently affected site (42, 43).

The reported frequency of CNS disease in BS children ranges from 5 to 15%, and it may be parenchymal or non-parenchymal. The former may present as an encephalomyelitis or aseptic meningitis, while the latter is usually characterized by intracranial hypertension secondary to venous sinus thrombosis (44, 45).

Gastrointestinal manifestations are represented by diarrhea, abdominal pain, and ulceration of the ileum, cecum, and colon and appear to be more common in Japanese patients compared with Turkish patients (15).

Due to the heterogeneity of disease and the lack of a diagnostic test, several autoinflammatory disorders with onset in childhood may exhibit BS-like symptoms. Periodic fever, aphthous stomatitis, pharyngitis, and cervical adenitis (PFAPA) syndrome, may be considered in cases of early or incomplete BS presentation (46). Mevalonate kinase deficiency (MVKD) and cyclic neutropenia may also be associated with recurrent mucosal ulcers. A young age of onset and the presence of prominent fever attacks may suggest MVKD diagnosis (47). Another rare monogenic disorder, A20 haploinsufficiency (HA20) can be misdiagnosed as BS. HA20 patients show Behçet-like phenotypes, including oral and genital ulcers, pathergy phenomenon, vascular thrombosis, as well as neurological and gastrointestinal involvement. Disease onset during early childhood, autosomal dominant inheritance, recurrent fever episodes and the presence of autoantibodies or autoimmune features favors the diagnosis of HA20 (48). Furthermore, BS has been increasingly recognized in association with constitutional trisomy 8 mosaicism and acquired trisomy 8 in adults. *RELA*-truncating mutation can also result in BS-like recurrent mucocutaneous lesions and neuromyelitis optica. Activation of the NF-κB pathway may underlie the BS-like phenotype in both the disorders (49). Therefore, the early presentation (<5 years of age), a strong family history and/or incomplete or atypical clinical features of BS should be considered as “red flags” to include the screening for monogenic autoinflammatory diseases in the diagnostic workup.

BS treatment largely depends on the site and severity of involvement. While consensus recommendations for adult BS were established by EULAR in 2018, there is a lack of consensus on management guidelines for pediatric BS due to its rarity, the heterogeneity of disease expression, and the absence of randomized controlled trials in this population (24). Consequently, management strategies have been largely extrapolated from adult studies and cohorts. Topical corticosteroids followed by colchicine have been considered as first-line treatment for oral and genital ulcers in both adults and children with BS. Disease-modifying anti-rheumatic drugs (DMARDs) such as azathioprine, mycophenolate mofetil, methotrexate, or sulphasalazine have been adopted as step-up treatment depending on the extent of organ and systemic involvement. In refractory cases or in case of severe organ involvement, biological agents like anti-TNFa drugs, anti-IL-1 (e.g., anakinra or canakinumab), and anti-IL-6 receptor (tocilizumab) have been employed (17).

Similarly to therapeutical approach, the current outcome measures such as disease activity, QOL and treatment adherence scores are not specifically tailored for pediatric BS patients. Leeds BD-QOL questionnaire has been validated in different studies and ethnic groups in adult BS patients, while none of the available tools have been designed for the pediatric population (14).

Disease activity scores and severity indexes tend to be lower in children as compared to adults, although this might be attributed to a shorter disease course rather than actual quiescent disease activity (13).

In conclusion, pediatric-onset and adult-onset BS share several epidemiological, clinical and therapeutic features, but the two conditions do show some peculiarities in each of these matters, which are summarized in Table 1.

**Table 1:** Peculiarities of pediatric onset-Behçet’s syndrome and adult onset-Behçet’s syndrome.

	Pediatric-onset BS	Adult-onset BS
Epidemiology	Rare disease Equal frequency among genders Higher prevalence in regions along the ancient Silk Road Familial clustering with genetic anticipation: screening for monogenic autoinflammatory disorders showing strong family history and BS-like features is advisable	Rare disease Equal frequency among genders Higher prevalence in regions along the ancient Silk Road
Clinical history	Relapsing–remitting course Frequent incomplete phenotype at onset Low frequency of genital ulcers Prevalence of ocular lesions vary according to age Ocular disease is more frequent in boys Main site of thrombosis: cerebral sinus veins	Relapsing–remitting course Higher burden of disease early after onset Oral and genital ulcers are the most frequent manifestation Main site of venous thrombosis: upper and inferior limbs
Classification	PED-BD set of classification criteria	ISG/ICBD set of classification criteria
Therapy	No consensus on management guidelines	EULAR recommendations

BS, Behçet’s syndrome.

## Transitional care in rheumatic diseases and Behçet’s syndrome

Transition concept does not refer to a one-time administrative event of transfer from pediatric to adult providers, and it does not have medical relevance only (2). The Society of Adolescent Health and Medicine Transition defined transitional care (TC) as the “purposeful, planned movement of adolescents and young adults (AYA) with chronic physical and medical conditions from child-centered to adult-oriented healthcare systems” (50). During adolescence and young-adulthood responsibilities gradually shift from parents/caregivers to patients themselves, so TC is meant to provide YP with the self-management skills and the access to the resources necessary to fulfill not only their traditional medical needs, but also their evolving psycho-social, educational and vocational needs, as they grow up. Therefore, transition is a dynamic and flexible process, and the planning of TC is driven by YP development, rather than the age at the time of transfer to adult services (2, 51). In facts, some authors have distinguished three stages of the process: (i) an initial phase of preparation beginning in early adolescence (2), (ii) a second phase in late adolescence focused on the event of transfer, and (iii) a third and final phase of variable length which follows the young patient as he/she gradually undertakes the adult services (51, 52). This may suggest that patients with child-onset BS should begin transition process at the time of diagnosis, since the disease usually occurs between 5 and 12 years old (11).

The importance of TC in YP with child-onset RD has been increasingly acknowledged and to date the key principles and the approach to TC have been embraced by several authors and consensus (5, 51, 53–55). In addition, in 2016 EULAR/PReS published the first set of standards and recommendations for the TC of YP with juvenile-onset RD (2). The expert panel agreed that YP should have access to high-quality, holistic and multi-disciplinary TC, in partnership with parents/caregivers and at least one pediatric rheumatologist and one adult rheumatologist, who together constitute the main participants of the transition process. Direct and honest communication between them is needed (2, 51, 55), and ideally there should be a combined meeting between YP and their family, the pediatric and the adult healthcare provider before and after the actual transfer. Other ideal participants of the process are nurses, psychologists, occupational therapists, social workers and, more importantly, primary care physicians as they contribute to the continuity of care during the period of transfer (2, 54). In a UK Delphi-study by Shaw et al. (55) where the “best practice” in TC was assessed as perceived by both providers and users (in this case, YP with JIA and their parents), continuity was seen by users as an important prerequisite for the following independency of the child from their parents.

Each health care provider involved in transition process must be trained on childhood-onset RD and the impact on YP, adolescence health issues, and communication with YP and their families. They must promote healthy lifestyle, self-management and shared decision making, efficiently addressing emotional, mental health and social issues of YP (2, 51, 54, 55).

Furthermore, EULAR/PReS also recommend that the multidisciplinary team involved in TC develops and agrees on a written and regularly updated transition policy, in cooperation with YP and their families as equal partners (2). Policies and protocols must establish the components of the multidisciplinary team and the designated coordinator, the transition timing, the adult services involved (e.g., rheumatologists, oculists, neurologists, etc.), an individualized transitional program (2, 54, 55) and the production of a written handover document (2, 51, 54, 55).

TC programs are inspired by the concept of resilience rather than by a pure medical model (54, 56), which means that YP need to acquire the knowledge and the range of competencies necessary to cope with the disease, and become fulfilled and independent adults in spite of the disease. In YP with BS, knowledge about basic aspects of diagnosis and prognosis could be an example, as well as information about drugs, regimens and possible adverse events, signs of a disease exacerbation, etc. Health professionals need to consider that knowledge promotes self-awareness and empowerment, hence they should educate YP and their families by delivering the tools and sources to have free access to reliable, accurate and up-to-date information (54, 55).

Nonetheless, to be effective, disease education must encompass also the aspects involving the real life of young people today. For instance, BS patients must be aware of the impact of smoking cigarettes on the occurrence of oral ulcers, the impact of the disease and therapies on reproductive health, implications of tattooing and piercing, and more (54). Another key skill to learn is to see health professionals independent of parents, and to contact them by themselves to seek medical advice, book appointments, ask for prescriptions, and more. This skill is part of other more general abilities which relate to confidence, self-advocacy and negotiation, and it was identified as an improving element for QOL of with JIA (54). Self-management skills are important and result from the awareness of one’s own health-status and disease impact. In BS they may involve the management of pain and concurrent infections, and self-medication practices such as initiating topic treatment for oral ulcers, regularly monitoring laboratory exams while assuming immunosuppressants, etc.

Finally, parents/caregivers are crucial and integral to TC and any transition program must address the needs of parents as well as young people (54). Among others, they need education about the impact of the disease on adolescents’ development, and clarification about their role in the transition process (51). In facts parents are important facilitators of transition, and it has been reported that family bond is essential in the resilience of YP with chronic illness (55), but overprotectiveness may be a problem during transition of their children (51).

### Transitional care as a challenge

The goal of TC is “to maximize lifelong functioning and potential through the provision of high-quality, developmentally appropriate healthcare services that continue uninterrupted as the individual moves from adolescence to adulthood” (2). To achieve this, TC focuses on enhancing patient’s social QoL. This transition marks a significant shift from childhood dependence to greater autonomy across various social spheres. As individuals move from adolescence to adulthood, they encounter increased opportunities to engage in diverse activities and contribute to society. This developmental progression encompasses emotional, cognitive, physical, and sexual domains and typically continues until at least midway through the third decade of life providing individuals with essential skills for successful adult navigation (57). However, there is some concern that adolescents and young adults (AYA) with pediatric-onset RD may face challenges in finding an employment and establishing relationships, as they might be unable to participate in the events and activities essential for social independence, despite receiving appropriate medical care (2, 5, 58–60).

“Transfer” is often understood as a singular event marking the shift in care responsibility from a pediatric provider to an adult one. In contrast, the term “transition” refers to a holistic process that starts well in advance of the actual transfer and continues into young adulthood, involving multiple stages, as mentioned above (2, 51). Transitioning children with RD to adult rheumatology services is essential to ensure a proper medical care, as pediatricians may be unfamiliar with adult-specific complications and with the evolving pathophysiology of the underlying disease with aging (61).

Although there is no consensus regarding the optimal timing to start transition, assessing the readiness of YP before starting this process appears crucial to ensure its success. Different questionnaires have been developed for this purpose, even if a standard tool has not been identified and a comparative analysis of these assessments has not been conducted (58, 60, 62, 63). A valuable example of readiness tool is given by the non-disease-specific Transition Readiness Assessment Questionnaire (TRAQ), which was developed with older adolescent patients. The main concerns about this tool are related to the predominantly medically orientation, in contrast to the more holistic evaluation of other tools such as the revised ON TRAC questionnaire (51).

In addition, TC should take into account cultural differences, and readiness surveys should be culturally sensitive (e.g., parents may be questioned in terms of education and employment status). In this regard, validation studies across different languages and cultural contexts are needed. However, the mere questionnaire results may not be reliable in establishing patient readiness, as many unpredictable factors can influence the transition process in real-life. Moreover, these tools are typically designed to evaluate readiness across a spectrum of chronic diseases, thus potentially overlooking the unique needs and challenges faced by patients with pediatric BS. Consequently, it remains unclear which questionnaire is best suited for assessing readiness to TC in BS AYA, as the domains assessed by the questionnaire may vary in their significance for transition and their optimal cutoff values remain undetermined. Therefore, developing assessment measures specifically tailored to the expectations of BS AYA could prove more beneficial (5, 61, 63).

Additionally, if it became evident that the holistic process needs to be tailored on the person, it should also be stated that a transitional process for a child with BS cannot be the same of a child with JIA. Consistent and appropriate tools need to be tested and validated for each specific disease on the way of the TC.

Another challenge is that the majority of BS cases present during adolescence, making it difficult to prepare AYA with BS for transition shortly after diagnosis. The transition program may be ignored by both clinicians and patients while the BS patient is still adapting to many new conditions, including the presence of a chronic disease and medications. Additionally, BS YP may develop altered physical appearances due to disease manifestations or medication side effects, alongside physical limitations, frequent hospitalizations and medical visits (5, 59, 60, 64). These factors may prevent their educational success and the life goals achievement. Moreover, this age group often prioritizes short-term interests over health considerations, with minimal regard for future-oriented consequences. Young adulthood is characterized by numerous changes within a brief timeframe, such as starting or finishing school, changing jobs, relocating for employment or university, as well as other significant life events like pregnancy, any of which can take the BS patient in unfamiliar and precarious circumstances.

As individuals with BS mature, they tend to acquire a deeper understanding about their condition, they demonstrate increased autonomy in managing their health, and develop more explicit vocational aspirations. An effective communication with BS AYA patients requires specific skills, such as asking parents to step out before addressing sensitive social history topics and exploring various aspects of adolescent social history, including career plans and peer influences (57). In this context, having psychologists on the transition team who are familiar with the cultural background of the country may facilitate discussions on these matters as cultural taboos may inhibit discussions about sexuality, and parental reactions may hind conversations about tobacco, alcohol, and drug use (57, 61, 63).

Disease activity constitutes another critical factor in the transition process. Transition is ideally recommended during a period of stable disease (2). However, available data indicate that a transfer during stable disease is provided in almost one-third of chronic RD YP (61, 63). The primary limiting factor is the unavailability of certain effective drugs not approved for the pediatric age and the subsequent restrictions on drug prescriptions. In the case of pediatric-onset BS, there are no comparative trials evaluating different therapeutic approaches and most management strategies are extrapolated from adult cases (49). Therefore, many treatments are employed off-label for this condition, leading to substantial challenges in drug accessibility. As a result, pediatric rheumatologists often prefer to transfer patients with active disease requiring these treatments directly to the adult providers, rather than waiting for disease remission.

Another challenge stems from the scarcity of data on TC in developing countries since most studies have been conducted in developed countries (61). Moreover, inadequate resources are cited as the primary barrier to implementing transition programs even in developed countries, therefore the current economic challenges in developing countries may further lead clinicians to underestimate the importance of non-emergency health services like TC. At this regards, nationwide transition policies are needed to establish customized programs that should incorporate multidisciplinary teams and rely on the unique characteristics of healthcare centers and individual patients (2, 5, 64).

Despite well-structured transition programs, success rates in AYA with RD are below 50%, with 10% of patients failing to continue follow-up after the first adult rheumatology visit (61). Cultural disparities between adult-oriented and pediatric care, including expectations on self-management, decreased involvement of family members, fewer on-site ancillary services, and stricter clinic rules and policies may discourage BS AYA from trusting the new adult clinic model, increasing the risk of gaps in continuity of care and poor disease outcomes (63). Consequently, collecting the patient’s feedback after the first transfer visit is crucial to ensuring the adequacy of adult rheumatology follow-up.

In conclusion, the design of a personalized transition program for YP with BS appears essential to ensure continuity of care and disease management and to prevent poor disease outcomes.

### Transitional care as an opportunity

The interest of rheumatology community for TC has grown in the past years, also following the increasing evidence supporting the benefits of an effective TC process. Recently, Bitencourt et al. (5) reviewed the outcomes of transition interventions in generic pediatric-onset chronic illnesses, reporting high satisfaction of patients, adherence to appointments schedules, improved rates of hospitalization, decreased death and greater cost-effectiveness. Regarding pediatric-onset RD, the first evaluation of any evidence-based TC program was conducted in a UK multicentre trial in JIA that showed significant 6 and 12 months improvement of perceived QOL, satisfaction, and knowledge, compared to baseline (52). In addition, other authors reported overall improvement for YP with RD in relation to follow-up, QOL, self-management, disease-specific knowledge, and physical and psychosocial status (65–68). Still, further research in this field is necessary, not to mention that data involving TC outcomes in BS is once again lacking.

In general, it is still not clear whether meeting the TC standards and goals leads to improved outcomes in RD, and there is no gold standard outcome measure to determine the success or the failure of a specific transition program (2, 5, 51, 69, 70). The first international consensus-based proposal regarding TC outcomes included structural parameters (coordination between healthcare professionals involved in the process, identification of the adult provider to take on the patient before transfer), and individual parameters (starting transition process early, discussing with patients about self-management, consider patient’s preferences to the planning of transition) (69). Moreover, the panel experts involved in the study only identified one essential indicator of successful transition, that is continuity of care (patient not lost at follow-up). Furthermore, in 2016 another interdisciplinary panel, also including YP, identified QOL as the most important outcome of TC by a Delphi-process (71). The transition process is critical to ensure that YP with chronic diseases obtain the best QOL possible as adults. The following major outcomes were also defined: (a) knowledge regarding the condition and the medication, (b) self-management skills and adherence to medication, (c) health services outcomes (e.g., attending medical appointments and low rates of unnecessary hospitalization), and (d) one social outcome (having a social network). These findings echo the healthcare transition model developed by Betz and colleagues (72), which is based on the concept that transition involves not only health service domains, but also individual and social domains. Nonetheless, vocational and psychological outcome measures appear to be less proposed by researchers (70, 71), in favor of the aforementioned traditional healthcare utilization and clinical outcomes, even in SLE and JIA (3, 73–80). In conclusion, multi-dimensional outcome measures are desirable in the evaluation of TC programs in all child-onset chronic diseases, including RD. These will contribute to design tailored transition plans, to promote continuity of care and ensure a high-quality relationship between the parties. In facts, outcome measures and indicators of effectiveness should be directed to both YP and physicians, since they both may limit the efficacy of the transition process (66, 81, 82). In particular, physicians treating YP with BS will learn to better address adolescents’ health issues within a rare and high-burden disease, adopting a person-centred approach focused on the young person as equal partner in the transition process. The resulting engagement and growing empowerment of the young patient and his/her family will contribute to build a mutual trust relationship which is essential for shared decision-making, disease-specific education and adherence.

Families and caregivers also should be taken into great account by health providers, since they also can represent a limiting factor for an efficient transition process. For example, parents may be overprotective of their children, and their transition readiness should also be evaluated before the transition process begins. For this purpose, a specialist nursing care during transition has been proposed.

Effectiveness of transition also relies on the resources delivered by health systems. Indeed, coordination, communication, consensus and continuity are challenging for healthcare systems and this is particularly relevant for chronic multi-system vasculitis like BS, which require coordination among several specialty clinics (51). EULAR/PReS panel experts defined transition as “resource consuming” and highlighted that without institutional funding transitional services and interventions cannot become a normal part of YP healthcare (2).

It is clear that transition provides challenges on a professional, clinical, individual and system level, but challenges may become opportunities if healthcare systems truly commit to promote developmentally appropriate care through suitable policies and investments, in response to the recent World Health Organization call to build youth-responsive health systems (83).

## Conclusion

Transition is a variable and multi-dimensional process inspired by a patient-centred approach, which attempts to holistically address health and psycho-social needs of YP with chronic diseases, while providing disease-specific education and self-management skills throughout their journey towards adulthood.

TC is a lacking research field in BS literature, and scarce experience of physicians and YP involving evidence-based transition programs is reported. Children and adolescents with BS are at risk of loss to follow-up, lack of adherence and poor prognosis since they grow by living with a chronic condition highly affecting their social and vocational needs. TC constitutes an enormous opportunity to ensure continuity of care and improve overall disease outcomes in these patients. Appropriate education of health professionals, a mutual trust relationship amongst the stakeholders, and suitable investments and policies by healthcare systems may overcome the numerous challenges of a high-quality and effective transitional care.

## Data Availability

The original contributions presented in the study are included in the article/supplementary material, further inquiries can be directed to the corresponding author.
